# Attenuating posttraumatic distress with omega-3 polyunsaturated fatty acids among disaster medical assistance team members after the Great East Japan Earthquake: The APOP randomized controlled trial

**DOI:** 10.1186/1471-244X-11-132

**Published:** 2011-08-16

**Authors:** Yutaka Matsuoka, Daisuke Nishi, Naoki Nakaya, Toshimasa Sone, Kei Hamazaki, Tomohito Hamazaki, Yuichi Koido

**Affiliations:** 1 Department of Psychiatry, National Disaster Medical Center, 3256 Midoricho, Tachikawa 190-0014, Japan; 2 Clinical Research Institute, National Disaster Medical Center, 3256 Midoricho, Tachikawa 190-0014, Japan; 3 Department of Adult Mental Health, National Institute of Mental Health, National Center of Neurology and Psychiatry, 4-1-1 Ogawahigashi-cho, Kodaira 187-8553, Japan; 4 Clinical Research Track Program, Translational Medical Center, National Center of Neurology and Psychiatry, 4-1-1 Ogawahigashi-cho, Kodaira 187-8551, Japan; 5 CREST, Japan Science and Technology Agency, 3256 Midoricho, Tachikawa 190-0014, Japan; 6 Department of Nutrition and Dietetics, Faculty of Family and Consumer Sciences, Kamakura Women's University, 6-1-3 Ofuna, Kamakura 247-8512, Japan; 7 Department of Rehabilitation, Faculty of Health Science, Tohoku Fukushi University, 1-8-1 Kunimi, Sendai 981-8522, Japan; 8 Department of Public Health, Faculty of Medicine, University of Toyama, 2630 Sugitani, Toyama 930-0194, Japan; 9 Department of Clinical Sciences, Institute of Natural Medicine, University of Toyama, 2630 Sugitani, Toyama 930-0194, Japan; 10 Head Office, Japan Disaster Medical Assistance Team, 3256 Midoricho, Tachikawa 190-0014, Japan

## Abstract

**Background:**

On March 11, 2011, a magnitude 9.0 earthquake, the most powerful ever recorded in Japan, and a massive tsunami struck off the coast of the Sanriku region. A Disaster Medical Assistance Team, a mobile medical team with specialized training that is deployed during the acute phase of a disaster, was dispatched to areas with large-scale destruction and multiple injured and sick casualties. Previous studies have reported critical incident stress (i.e. posttraumatic stress disorder symptoms and depressive symptoms) among rescue workers as well as the need for screening and prevention for posttraumatic stress disorder. So far we have shown in an open trial that posttraumatic stress disorder symptoms in critically injured patients can be reduced by taking omega-3 fatty acids intended to stimulate hippocampal neurogenesis.

**Method/Design:**

This study is designed to determine the effectiveness of attenuating posttraumatic distress with omega-3 polyunsaturated fatty acids among Disaster Medical Assistance Team members after the Great East Japan Earthquake, and is named the APOP randomized controlled trial which is currently ongoing. First, we will provide psycho-education on posttraumatic distress, which is common in responders to the Disaster Medical Assistance Team members deployed to the disaster area. Second, observational research will be conducted to evaluate critical incident stress following the completion of medical activities. Third, team members who provide consent to participate in the intervention research will be randomly divided into a group given an omega-3 fatty acid supplement and a group not given the supplements. Outcome will be evaluated at 12 weeks after the supplements are shipped to the team members.

**Discussion:**

Measures that address critical incident stress in disaster responders are important, but there is no substantial evidence that links such measures with prevention of posttraumatic stress disorder. Thus, any confirmation through this study that the intake of omega-3 fatty acid supplements serves as a simple preventative measure for critical incident stress will be of great significance.

**Trial registration:**

UMIN Clinical Trials Registry, UMIN000005367

## Background

On March 11, 2011 at 14:46, a magnitude 9.0 earthquake, the most powerful ever recorded in Japan, and a massive tsunami struck off the coast of the Sanriku region, leaving over 20,000 dead or missing. The earthquake and subsequent tsunami, now known as the Great East Japan Earthquake, was the worst disaster Japan has experienced since World War II, causing psychological trauma among the survivors as well as critical incident stress among the rescue workers.

Even veteran responders with medical expertise deployed to disaster areas may experience significant psychological effects from exposure to the tragic circumstances they witness. More specifically, they may experience a variety of psychological reactions, including irritability, difficulty sleeping, feeling that the situation and work at the disaster site are unreal, recounting disaster efforts, nightmares, avoidance or reluctance to talk about people and objects that trigger memories of disaster areas, feelings of powerlessness in being unable to do anything, strong feelings of self-reproach, and anger. In fact, a study on the effects of critical incident stress in firefighters found an association between work-related psychological trauma and the onset of posttraumatic stress disorder (PTSD) [1]. Moreover, in a study of 355 medical care personnel sent to aid trauma victims of an airline crash, 13.5% developed PTSD within 18 months of the crash [2]. Similarly, in a study of 207 aid workers deployed to the site of the September 11 terrorist attack in New York in 2001, 16.7% developed PTSD and 21.7% developed depression at 13 months after the attack [3]. Appropriate evaluation of critical incident stress and screening and prevention of secondary psychiatric illness are thus crucial tasks.

In the pathogenesis of PTSD, fear memories become excessively consolidated and extinction learning does not progress [4]. Kitamura recently found that the period of hippocampus-dependent fear memory is longer in mice with decreased hippocampal neurogenesis and shorter in mice with active hippocampal neurogenesis [5], indicating that the level of hippocampal neurogenesis is a crucial factor in determining the period of hippocampal-dependent fear memory. This finding suggests that the fear memories characteristic to PTSD may be controlled by aptly regulating hippocampal neurogenesis [6]. We are currently conducting a randomized controlled trial different from the one described herein to investigate the preventive effectiveness of omega-3 fatty acids for preventing PTSD in physically injured patients (ClinicalTrials.gov Identifier: NCT00671099) since these fatty acids have been confirmed to enhance hippocampal neurogenesis in animal studies [7,8]. The open preliminary trial found that post-trial PTSD symptoms were significantly alleviated in injured patients who took the omega-3 fatty acids [9].

The present study aims to determine the effectiveness of attenuating posttraumatic distress with omega-3 polyunsaturated fatty acids among Disaster Medical Assistance Team (DMAT) members who are deployed during the acute disaster phase following the Great East Japan Earthquake. This study named the APOP randomized controlled trial aims to (1) provide psychoeducation on posttraumatic distress common among rescue workers to DMAT members dispatched to disaster areas, (2) assess critical incident stress among the DMAT members following completion of their medical duties, and (3) recruit these DMAT members to a 12-week study investigating the effects of omega-3 fatty acids in reducing stress, with consenting participants randomly allocated to either an omega-3 acid fatty acid supplementation group or a non-supplementation group. The efficacy of omega-3 fatty acids in reducing critical incident stress (PTSD symptoms or depressive symptoms) at 12 weeks will be examined.

## Methods/Design

### Study Design

The present study is a randomized clinical trial that will compare an intervention group that receives psychoeducation and omega-3 fatty acid supplementation with a parallel control group that receives psychoeducation only.

### Participants

The DMAT service was established by the Ministry of Health, Labour and Welfare of Japan in April 2005 and operates from the Disaster Medical Center of the National Hospital Organization. DMAT members are physicians, nurses, and operational coordination staff (medical or clerical staff who are neither physicians nor nurses) who are dispatched as a mobile medical team with specialized training that is capable of acting during the acute phase (roughly within 48 hours) of a large-scale disaster and in the event there are multiple injured or sick casualties. Following the Great East Japan Earthquake, DMAT activities commenced on the same day, namely March 11, and concluded on March 22. Recruited DMAT members deployed to the disaster area met the following inclusion criteria: 1) aged 18 years or older; 2) a native Japanese speaker or non-native speaker with Japanese conversational abilities; and 3) physically and psychologically capable of understanding and providing consent for study participation. The exclusion criterion was regular intake of warfarin for at least 3 months before deployment.

### Estimation of Sample Size

The required sample size for intervention research was estimated at 48 cases per group. Based on our previous research [9,10], we estimated that the mean of improvement in the Impact of Event Scale-Revised (IES-R) score as a primary outcome measure would be 10 (*SD *= 15) for the intervention group and 0 (*SD *= 15) for the non-intervention group. We set α level at .05 and β at .10. This brought us to our required sample size estimation of 48 cases per group. This study set case numbers above that required, with consideration given in its design for the following: the sample would be recruited from a population different from that of previous studies (i.e. medical assistance members); the control group would receive psychoeducation; and the actual participant number was estimated. Thus, we allowed up to 150 cases for the intervention group and 300 cases for the control group.

### Enrolment procedure

**Figure 1:**
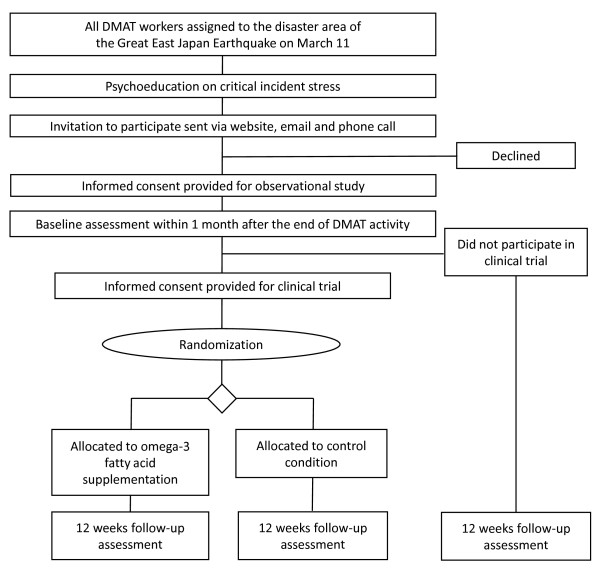
**Flow diagram of the study**.

The procedure for participant enrollment is shown in Figure 1. A written guide to the study was posted to the Emergency Medical Information System (EMIS) by the DMAT office and affiliated hospitals with DMAT members were notified of the posting by their local municipalities. The written guide contained a written explanation of the research, a consent form, a questionnaire for assessing critical incident stress, a leaflet on psychoeducation, and reference materials on the intervention research (a copy of a general medical journal article summarizing the preliminary trial and the original manuscript of the researchers [9]) uploaded to the EMIS website. All documents were then mailed to DMAT members and a mass email was sent to all DMAT members who had been deployed to the disaster area. In addition, the DMAT Office at each of the DMAT members' affiliated hospitals was called to request that members be encouraged to participate in the study. Because individual explanation to eligible members was difficult to provide, eligible members could take time to read the written documents on their own and provide consent by returning the informed consent form (by fax or mail). Consent for participation was confirmed for each of two stages of the study: the first stage was participation in only observational research to assess critical incident stress following deployment to the disaster areas; the second stage was participation in intervention research involving omega-3 fatty acid supplementation after the assessment of critical incident stress.

### Interventions

Omega-3 fatty acid supplementation in the intervention group

In line with previous research [11], participants are taking 7 capsules per day, each containing 320 mg of oil following return from their duties in the disaster area. The omega-3 fatty acid composition of each capsule is 70% docosahexaenoic acid and 7% eicosapentaenoic acid. Each capsule is placed in a brown 500-ml polyethylene container with a wide opening. Participants were instructed to take the capsules after eating and additionally told that they may take a full day's dosage at one time. Participants will be contacted to ensure regular capsule intake and safety monitoring at 2, 4, 8, and 12 weeks from the start of the intervention. Whenever inquiries are received from participants, necessary information will be provided to them.

### Control condition

A placebo capsule was not prepared. A leaflet on psychoeducation about posttraumatic distress focusing on critical incident stress was provided to participants and they will be contacted about their situation at 2, 4, 8, and 12 weeks. Whenever inquiries are received from participants, necessary information will be provided to them.

### Randomization

In regard to participant enrollment and group assignment, DMAT members who returned the informed consent form for the omega-3 fatty acid intervention research have been enrolled as participants. Central registration involved assigning participants to groups according to an assignment diagram developed by trial statisticians. Core investigators were single-blinded, and participants were randomly allocated to either the omega-3 acid group or the control group using block randomization. The participants were stratified by sex, and randomization was conducted by permuted block method using a four-person block. Sex was an adjustment factor, as previous studies show that the prevalence of PTSD and major depressive disorder are higher in women than in men [12]. Omega-3 fatty acid capsules were individually shipped to the address designated by each participant following random assignment. All information on case assignments will be disclosed after the final follow-up on participants has been completed and all data secured.

### Informed Consent

The following describes the explanation and information used in obtaining consent from eligible participants. Participation in the study is voluntary. There is no penalty for declining to participate. Participants may withdraw from the study at anytime without penalty. Information provided covered the following areas: reasons for selection, the names and occupational titles of the researchers, the meaning, purpose, method, research period, expected benefits of participation, protection of privacy, possibility of patents from the study, possible risks or unpleasant physical adverse effects, disclosure of results related to the study, the publication of results without participant identifiers, possible risks, affiliated organizations of researchers and their relationships with the organizations, methods of data use, and period of data preservation.

### Baseline Assessment

#### Basic information on the participants

Basic information obtained from the participants comprised name, sex, age, hospital affiliation, contact information, e-mail address, height, weight, occupation, marital status, number of children, highest education completed, smoking and drinking habits, years of experience, experience of deployment to disaster areas, use of omega-3 supplements, dietary habits, physical illness, and previous history of physical and psychiatric illnesses.

#### Information about traumatic events

Participants were surveyed about the following items, in addition to those variables identified as risk factors for PTSD in previous research [2,13]: period of deployment, stress prior to deployment, injury during deployment, experience of saving a child during deployment, experience of contact with corpses, fears of radiation, duration of time spent watching earthquake news reports, and current subjective physical symptoms.

#### Peritraumatic Distress Inventory

The Peritraumatic Distress Inventory (PDI) is a 13-item questionnaire, developed by Brunet et al [14], for quantification of fear and sense of helplessness in the trauma cycle (the period during and directly after a traumatic experience). Previous studies have shown that one set of the PDI items predict PTSD symptoms [15]. With permission from creators Brunet and Marmar, we previously created the Japanese version and confirmed its validity and reliability [16,17].

#### Impact of Event Scale- Revised

The Impact of Event Scale-Revised (IES-R) is a self-reporting questionnaire about PTSD symptoms that was developed in the U.S. It is the most widely used measure internationally in all forms of disaster-area research [18]. The IES-R is composed of 22 items on the three largest symptoms in the diagnostic criteria of PTSD, namely re-experiencing, avoidance, and increased physiological arousal. Respondents rate symptoms experienced in the previous week. The validity and reliability of the Japanese version of the IES-R has been confirmed [19].

#### The Center for Epidemiologic Studies Depression Scale

The Center for Epidemiologic Studies Depression Scale (CES-D) is a self-reporting questionnaire on depression that was developed by Radloff et al [20]. The higher the total score is to the maximum score of 60, the more severe the depressive state. The cut-off score for a mood disorder is considered to be 16 points. Validity and reliability of the Japanese version have been confirmed [21].

#### Kessler K6 Scale

The Kessler 6 Scale (K6) is a self-reporting questionnaire designed to effectively screen for psychiatric disorders and mood and anxiety disorders, where respondents rate their condition for the last month [22]. Validity and reliability of the Japanese version has been confirmed [23]. An adequate cut-off score on the K6 for serious mental illness is 0-12 vs. 13 or more [24].

#### Resilience Scale and Resilience Scale-Short Version

The 25-item Resilience Scale (RS) and its shortened 14-item version (RS-14) are self-reporting questionnaires developed by Wagnild and Young for quantitative evaluation of resilience [25]. Among European and U.S. scales for resilience, its reliability and validity are considered the most established. We created Japanese versions of the RS and RS-14 with the permission of Wagnild and confirmed their reliability and validity [26]. The present study used the short RS-14 version.

### Follow-up Assessment Schedule

The overall procedure of the trial is shown in Figure 1. Follow-up assessment schedule from baseline to 12 weeks is shown in Table 1.

**Table 1:** Summary of outcome measures of the APOP clinical trial

Primary Outcome	Baseline	2 weeks	4 weeks	8 weeks	12 weeks
IES-R	X				X

Secondary Outcomes					

CES-D	X				X
K6	X				X
RS-14	X				X

Safety monitoring		X	X	X	X

Determinants					

Demographic	X				
Life style	X				
Past medical history	X				
Detailed information about disaster-related event	X				
PDI	X				

### Outcomes

#### Primary outcome

The total score on the IES-R at 12 weeks after shipment of the supplements is the primary outcome measure.

#### Secondary outcomes

Total scores on each of the CES-D, the RS-14, and the K6 at 12 weeks after shipment of the supplements constitute the secondary outcome measure.

### Safety Management and Study Monitoring

Safety of the intervention is evaluated by the presence of an adverse event during the observation period. The investigators will contact the participants regarding the presence of any adverse events at 2, 4, 8, and 12 weeks after the start of the omega-3 fatty acid supplementation intervention. When an adverse event occurs, the investigators will rate the degree of the event as either "mild", "moderate", or "severe".

The principal investigator will assess the circumstances surrounding the occurrence of a serious adverse event and/or an event that may affect the future of the investigation. Cases will be reported to an independent data safety monitoring board and the company providing the trial capsules, and related information will be shared with them. The blinding of cases may be discontinued as deemed necessary and information gathered so that the causes behind the occurrence may be investigated. The ethics committee of the facility may also be notified.

The investigation will cease when (1) discontinuation of the study is recommended by the data safety monitoring board due to an adverse event or side effect that makes continuation of the investigation difficult or (2) the principal investigator decides not to continue implementation.

### Statistical analysis

All analyses were conducted according to the intention-to-treat principle. Analysis of covariance (ANCOVA) will be used to obtain differences between the means, 95% confidence interval values, and P values. Covariates for ANCOVA are sex, age, and IES-R scores at baseline. A two-tailed test will be used, with the α level set at .05%. Evaluation by regression models will be conducted as necessary. Validity of the results will be evaluated through sensitivity analysis and filling missing data.

Analysis of the secondary outcome measure will be conducted to add to discussion of the results of the primary outcome measure. Adjustment will not be conducted for data duplication because secondary statistical analyses are exploratory. A two-tailed test will be used, with the α level set at .05%. Evaluation by regression models will be conducted as necessary. Validity of the results will be evaluated through sensitivity analysis and filling missing data.

### Time periods during the study

Research will be conducted from April 1 to September 30, 2011. Participant registration for observational research was from April 2 to 22, and participant registration for intervention research was from April 2 to 12. Follow ups are to be conducted after the omega-3 supplement shipments until August 31.

### Ethical Considerations

The present study protects the rights and welfare of participants in the spirit of ethical guidelines outlined under the Declaration of Helsinki. The study further respects the ethical principles of the Ministry of Health, Labour, and Welfare of Japan. Confidence can be assured in the ethics, safety, scientific rigor, and reliability of the research. Personal information obtained in the course of the research will be strictly secured to avoid external leaks. Because the study is a dietary intervention, no special compensation will be paid in the event of health damage directly related to the research. The research plan (2010-32) was deliberated upon and approved by the Ethics Committee of the National Disaster Medical Center on April 1, 2011.

## Discussion

Declines in physical and mental health due to critical incident stress in disaster aid workers or rescue workers has been demonstrated in previous research, but specific, adequate measures to counter critical incident stress have not been developed. The development of measures that can realistically be practiced by large numbers of aid workers is extremely important. Six years have passed since the DMAT service was established and little examination of critical incident stress among DMAT members has been conducted thus far. This study is designed to understand the phenomenon of critical incident stress among DMAT members and conduct the APOP clinical trial. The trial will provide omega-3 acid supplements to DMAT members stationed in all regions of Japan as a method to promote mental health without requiring individualized care from a mental health professional.

The use of self-reporting questionnaires while inferior to that of a clinical interview as an assessment of the APOP study research outcomes for PTSD and depressive symptoms, it is a reasonable assessment method given this type of emergency situation. We are currently implementing separate random comparative trials to prevent PTSD in physically injured patients (ClinicalTrials.gov Identifier: NCT00671099) and are evaluating PTSD through structured clinical interviews. The APOP study was designed at a time of crisis, 1 week after the earthquake occurred, and recruiting sufficient participants was considered difficult if a placebo group were to be used. Another limitation of the study is that fatty acid composition of red blood cell membranes could not be measured to confirm intake compliance of the omega-3 fatty acid supplements. With these limitations in mind, we do believe the results of the APOP clinical trial will be of importance: natural and man-made disasters occur across the globe and omega-3 fatty acid supplementation, if found to be efficacious for preventing critical incident stress, could contribute to maintaining the mental health of disaster relief workers in the future.

## Competing interests

Dr. Matsuoka has received research support from the Japan Science and Technology Agency, CREST, and the Ministry of Health, Labor, and Welfare of Japan and lecture fee from Eli Lilly Japan. Dr. Nishi has received research support from Toray Industries, Inc., and the Foundation for Total Health Promotion and lecture fee from Qol Co., Ltd. Dr. K. Hamazaki has received research support from the Japan Society for the Promotion of Science, the Tamura Foundation for Promotion of Science and Technology, and the Ichiro Kanehara Foundation for Promotion of Medical Sciences and Medical Care, and consultant fees from Polyene Project, Inc. and Otsuka Pharmaceutical Co., Ltd., and lecture fee from Nippon Suisan Kaisha, Ltd. Dr T. Hamazaki has received research support from the Japan Society for the Promotion of Science, Open Research Center for Lipid Nutrition (Kinjo Gakuin University), and Nippon Suisan Kaisha, Ltd., and consultant fees from Polyene Project, Inc. and Otsuka Pharmaceutical Co., Ltd., and lecture fees from Mochida Pharmaceutical Co., Ltd. Dr. Koido has received research support from the Ministry of Health, Labor, and Welfare of Japan, Ono Pharmaceutical Co., Ltd., Astrazeneca K.K., Bristol-Myers Squibb Company, and National Center of Global Health and Medicine. All other authors declare that they have no competing interests with this work.

## Authors' contributions

YM and DN conceived the study and drafted the original protocol. YM, DN, NN, TS, KH, TH, and YK participated in the refinements of the protocol. YM, DN, KH, and TH decided the content of the omega-3 fatty acid supplementation, YK managed the enrolment procedure and overall regulation of the trial, all authors contributed to the design of the study, and TS and NN calculated sample size and decided the analytic strategy. All authors read and approved the final manuscript.

## Pre-publication history

The pre-publication history for this paper can be accessed here:

http://www.biomedcentral.com/1471-244X/11/132/prepub
